# Redox Species of Redox Flow Batteries: A Review

**DOI:** 10.3390/molecules201119711

**Published:** 2015-11-18

**Authors:** Feng Pan, Qing Wang

**Affiliations:** Department of Materials Science and Engineering, National University of Singapore, Singapore 117575, Singapore; panfeng@u.nus.edu

**Keywords:** redox flow battery, redox species, energy density

## Abstract

Due to the capricious nature of renewable energy resources, such as wind and solar, large-scale energy storage devices are increasingly required to make the best use of the renewable power. The redox flow battery is considered suitable for large-scale applications due to its modular design, good scalability and flexible operation. The biggest challenge of the redox flow battery is the low energy density. The redox active species is the most important component in redox flow batteries, and the redox potential and solubility of redox species dictate the system energy density. This review is focused on the recent development of redox species. Different categories of redox species, including simple inorganic ions, metal complexes, metal-free organic compounds, polysulfide/sulfur and lithium storage active materials, are reviewed. The future development of redox species towards higher energy density is also suggested.

## 1. Introduction

To meet the energy demands for the increasing global population, the present energy production of the world will be doubled by 2050 [1]. Our society is more aware than ever before of sustainability issues. Due to limited fossil fuel resources and global climate change, the energy increase must be achieved without dramatic CO_2_ emissions [2]. Under this goal, renewable energy technologies have been popular topics in recent decades. Renewable energy sources, such as wind, solar and tidal, are all generally dispersed and inherently intermittent [3]. Due to the capricious nature of renewable power, advanced large-scale energy storage devices are required to make the best use of these renewable energy resources. However, the present energy storage capacity can only store around 1% of the energy consumed worldwide [1], and the current trend seems to be requiring more energy storage devices at the grid scale to increase the penetration of renewable energy.

**Figure 1:**
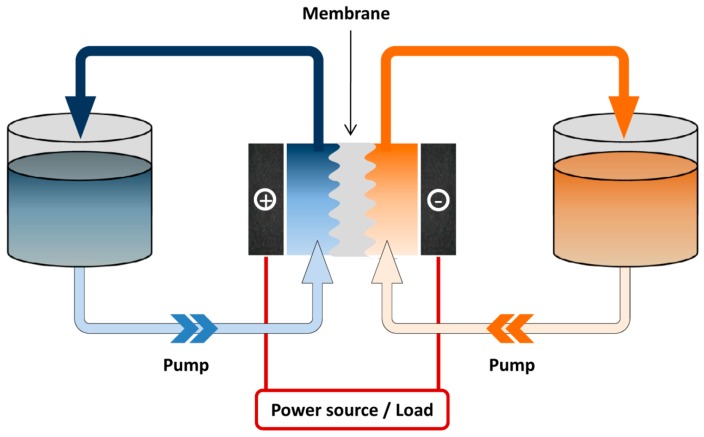
Schematic illustration of a redox flow battery. Active species are stored externally in the storage tanks, while the conversion between electrical and chemical energy occurs in the cell unit.

Rechargeable batteries offer an efficient way to store energy. Available battery technologies include lithium ion, nickel-metal hydride (Ni-MH), lead acid, redox flow and the sodium-sulfur (Na-S) system. Among them, the redox flow battery (RFB) is considered a promising energy-storage solution, which is suitable for stationary applications due to its modular design, good scalability, flexible operation and moderate maintenance cost [4]. RFB systems possess a unique structure, which consists of three parts: stack cell, energy storage tanks and the flow system (Figure 1). Unlike the enclosed configuration of lithium ion batteries where energy is stored with the electrode sheets, redox flow batteries employ active species dissolved in the liquid electrolytes, which are stored in the tanks, separated from the electrodes [5]. Upon operating, the fluids (catholyte and anolyte) containing redox active species are driven by the pump and circulate through each of the half compartments of the cells. The conversion between chemical energy and electrical energy occurs while the electrolytes are flowing through the electrodes [6]. Such a configuration brings RFB the most unique and attractive feature that other battery systems do not have: decoupled energy storage and power generation, which enables an independent control of capacity and power to meet different requirements ranging from a few kWh to several MWh.

The redox flow battery has been developed for large-scale application for decades, and some of the RFB systems have been successfully demonstrated at the megawatt scale. However, none of the present systems are widely commercialized. The most severe challenge for RFB is the low energy density, which results in a high cost for unit energy storage. For instance, the energy density of state-of-the-art vanadium-based RFB is in the range of 20–30 Wh·L^−1^, which is much lower than other battery systems, such as lithium ion batteries [7]. Moreover, compared to lithium ion batteries, whose energy density was enhanced by 8%–9% per year since the 1990s [8], the major systems in the RFB field have remained almost the same for more than two decades [9].

The redox active species is the most important component in redox flow batteries, which dictates the overall performance. From the perspective of energy density, the most important characteristics are redox potential and solubility. The cell voltage is determined by the equilibrium potentials of active species in the cathodic and anodic half-cells, while the capacity is dependent on the effective concentration, which is the solubility multiplied by the number of electrons transferred in the redox reactions [5,10]. Here, we provide an overview of the recent studies on redox species and the development of a redox flow battery. In traditional aqueous RFB systems, the redox species are mostly limited within the category of transition metal ions and halogen ions. In recent year, the non-aqueous system has become a hotspot, because it provides a wider electrochemical window, which further allows for more redox species, like various organic compounds, to be employed. Meanwhile, molecular engineering is being applied to modify those redox couples towards higher solubility and more suitable redox potential. In addition, there have been several new approaches combining RFB with the lithium ion battery concept.

We noticed that several comprehensive reviews have been published in the past few years. Ponce de leon *et al*. discussed the fundamental aspects of different kinds of RFB [11]. The reviews by Skyllas-Kazacos *et al*. and by Alotto *et al*. discussed the technology, application and commercialization status of RFB [4,7]. Zhang *et al*. reviewed the ion exchange membrane in a vanadium redox flow battery [12], while Shin *et al*. gave an overview of membranes for a non-aqueous flow battery [13]. A review by Weber *et al*. discussed the transport and kinetic behaviors on electrode reactions [6]. Leung *et al*. provided a summary of RFB chemistries, design considerations and modeling and applications [14]. Wang *et al*. gave a brief review of the features and progress of the Li-ion and redox flow battery systems and discussed the concept of the Li-redox flow battery [15]. More recently, Wang *et al*. reviewed the progress of RFB, including cell-level chemistry, electrodes and membranes [9]. Most of them are focused on cell-scale chemistry. Here, we present a review focused on the redox species of redox flow batteries. With an overview of different categories of redox species, a trend towards future higher energy density can be suggested.

## 2. Simple Inorganic Ions

The early research on the redox flow battery was mostly based on metal ions and halogen ions. The simple chemistry and high solubility makes metal ions the most popular candidates for aqueous redox flow batteries. On the other hand, halogens are intensively researched as cathodic active species owing to their high solubility, reversible redox reactions at high potentials and relatively low molecular weight. The most studied halogens, bromine and iodine, show redox potentials within the potential range of water electrolysis. The recent development of aqueous RFB involves a wider range of redox species. Most of them still fall in the category of transition metal ions and halogen ions, such as VCl_3_/VCl_2_, Br^−^/ClBr^2−^ [16], Ce^3+^/Ce^4+^ [17], Mn^2+^/Mn^3+^ [18], Ti^3+^/Ti^4+^ [19], Cu/Cu^1+^, Cu^1+^/Cu^2+^ [20,21], and others [22]. A summary of simple inorganic ions redox species is shown in Table 1. The concentrations displayed here are based on the demonstrated flow cells.

### 2.1. Iron/Chromium

The first modern redox flow battery was the Fe/Cr system primarily developed at NASA. The Fe^3+^/Fe^2+^ and Cr^3+^/Cr^2+^ redox couples were employed in the cathodic and anodic electrolytes, delivering a cell voltage of 1.18 V [23,24].

(1) $$\text{Cathodic: }Fe^{3+}+e^{-}↔Fe^{2+}\;0.77\text{ V }vs.\text{ standard hydrogen electrode (SHE)}$$

(2) $$\text{Anodic: }Cr^{2+}-e^{-}↔Cr^{3+}\;-0.41\text{ V }vs.\text{ SHE}$$

The Fe^3+^/Fe^2+^ redox couple shows good reversibility and fast kinetics. However, the slow kinetics of Cr^3+^/Cr^2+^ usually requires an elevated operation temperature, which significantly increases the cost [25]. Meanwhile, the low redox potential of Cr^3+^/Cr^2+^ may cause H_2_ evolution, which limits the Coulombic efficiency and cycle life.

### 2.2. All-Vanadium

The vanadium redox flow battery (VRB) was first proposed in 1980s by Skyllas-Kazacos and co-workers [26]. Vanadium has four valence states to form two redox couples, V^2+^/V^3+^ and VO_2_^+/^VO^2+^. VRB employs vanadium as the only element in both the catholyte and anolyte, giving a cell voltage of about 1.26 V in 2.0 M H_2_SO_4_ solution [27]. The single compound configuration controls the crossover contamination, which allows a long system life of about 15–20 years [28].

(3) $$\text{Cathodic:}{{\text{ VO}}_{2}}^{+}+2{\text{H}}^{+}+{\text{e}}^{-}↔{\text{VO}}^{2+}+{\text{H}}_{2}\text{O }1.00\text{ V }vs.\text{ SHE}$$

(4) $$\text{Anodic: }V^{2+}-e^{-}↔V^{3+}\;-0.26\text{ V }vs.\text{ SHE}$$

The all-vanadium flow battery is the most extensively-researched redox flow battery technology, and some VRB demonstration systems at the MWh scale have been installed [29,30,31]. The concentration of vanadium species is around 2.0 M in acidic aqueous electrolytes, and the energy density is 20–30 Wh·L^−1^. Although it seems to have advantages over other flow battery systems, such a low energy density makes it less attractive when compared to rivals, the like lithium ion battery, and this is also the biggest barrier for commercialization. Meanwhile, the toxicity hazard of soluble vanadium and the strong corrosive strength of VO_2_^+/^VO^2+^ also hinder the widespread adoption of VRB [32]. Various modifications have been proposed based on vanadium redox flow battery chemistry [33]. For instance, researchers from the Pacific Northwest National Laboratory (PNNL) reported a Fe/V battery, combining the advantages of the Fe/Cr system and VRB, while avoiding their drawbacks, such as the slow kinetics of Cr^3+^/Cr^2+^ and the high corrosive strength of VO_2_^+/^VO^2+^ [34]. Meanwhile, researchers from the University of Twente reported a new concept of a vanadium/air battery with V^3+^/V^2+^ as the anodic redox couple and H_2_O/O_2_ as the cathodic redox couple [35,36].

### 2.3. Vanadium/Bromine

The vanadium-bromine battery employs VBr_3_/VBr_2_ and Br^−^/ClBr^−^ in the anolyte and catholyte, respectively, with HBr and HCl as the supporting electrolyte [29].

(5) $$\text{Cathodic: }2Br^{-}+Cl^{-}↔ClB{r_{2}}^{-}+2e^{-}\;1.09\text{ V }vs.\text{ SHE}$$

(6) $$\text{Anodic: }2VBr_{3}+2e^{-}↔2VBr_{2}+2Br^{-}\;-0.26\text{ V }vs.\text{ SHE}$$

The higher solubility of vanadium bromine (1.0–3.0 M) than vanadium sulfate increases the energy density. One disadvantage of the vanadium/bromine system is the possible Br_2_ vapor emission [29].

**Figure 2:**
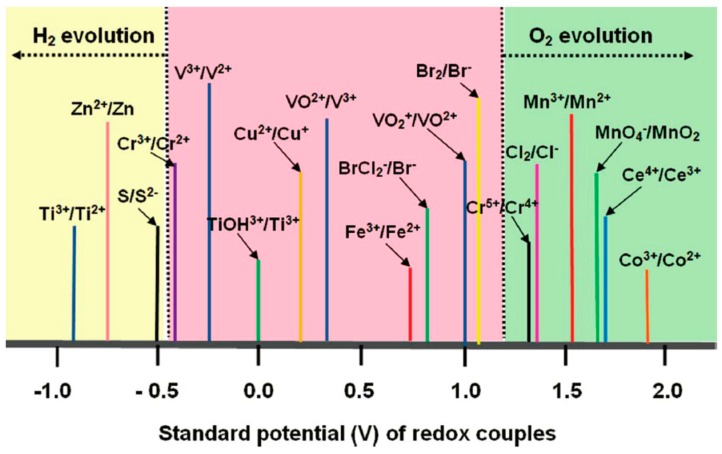
Redox potentials (*vs*. standard hydrogen electrode) of various redox couples (reproduced with permission from Chem Rev 111, 3577–3613 (2011); Copyright 2011, American Chemical Society).

### 2.4. Zinc/Bromine

The Zn-Br battery (ZBB) is another successful redox flow battery system, which has been demonstrated for decades [37]. The cathodic reaction involves the reduction of Br_2_ and oxidation of Br^−^, while Zn strips and plates onto the anode. ZBB is classified as a hybrid flow battery, in which one of the electrodes involves non-liquid reactants. The synergy between dissolved redox couples and metal anode usually brings great enhancement in energy density, because the volume of anolyte is eliminated.

(7) $$\text{Cathodic: }Br_{2}+2e^{-}↔2Br^{-}\;1.09\text{ V }vs.\text{ SHE}$$

(8) $$\text{Anodic: }Zn-2e^{-}↔Zn^{2+}\;-0.76\text{ V }vs.\text{ SHE}$$

ZnBr_2_ has high solubility over 2.0 M, but the charged species Br_2_ is less miscible in aqueous solvent, which substantially limits the achievable energy density of the Zn-Br battery. Moreover, the stability of ZBB is challenged by the corrosivity of Br_2_. Numerous redox couples have been studied to replace the bromine catholyte of ZBB [38,39,40,41] (Figure 2). Leung *et al*. investigated the Zn/Ce battery by employing Zn(CH_3_SO_3_)_2_ and Ce(CH_3_SO_3_)_3_ in CH_3_SO_3_H electrolyte [39]. The large redox potential between Zn and Cerium delivers a high open circuit voltage (OCV) of 2.40 V. Such a high voltage requires proper electrode materials and electrolyte additives to suppress the side reactions, which are mainly oxygen and hydrogen evolutions. Very recently, Li *et al*. reported a zinc-polyiodide battery with highly soluble ZnI_2_ electrolyte [42]. By utilizing an ambipolar electrolyte, where both cationic and anionic ions from a single soluble compound are both energy-bearing redox active species, the electrolyte eliminated the need for non-active counter ions, such as the Cl^−^ and SO_4_^2−^, commonly used in vanadium and Fe/Cr systems [43]. A high concentration ZnI_2_ of 5.0 M was demonstrated in the reported lab cell. The energy density is claimed to be as high as 166.7 Wh·L^−1^, which is comparable to lithium ion batteries. However, the high viscosity and I_2_ precipitation have hindered the practical application of the system.

**Table 1:** A summary of simple inorganic ion redox species. SHE, standard hydrogen electrode; SCE, saturated calomel electrode.

Redox Species	Demonstrated Concentration/mol·L^−1^	Redox Potential/V	Reference
VCl_3_/VCl_2_	1.0	−0.58 *vs.* SCE	[16]
Br^−^/ClBr_2_^−^	1.0	0.80 *vs.* SCE	[16]
Cl_2_/Cl^−^	1.0	−1.36 *vs.* SCE	[44]
Fe^2+^/Fe^3+^	2.0	0.77 *vs.* SHE	[23,24]
Cr^3+^/Cr^2+^	1.0	−0.41 *vs.* SHE	[23,24]
Ti^3+^/Ti^4+^	1.1	0.04 *vs.* SHE	[19]
V^3+^/V^2+^	2.0	−0.26 *vs.* SHE (2.00 M H_2_SO_4_)	[27]
VO^2+^/VO^2+^	2.0	1.00 *vs.* SHE (2.00 M H_2_SO_4_)	[27]
Zn/Zn^2+^		−0.76 *vs.* SHE	[27]
Br_2_/Br^−^	2.0	1.09 *vs.* SHE	[27]
Ce^3+^/Ce^2+^	0.5	1.67 *vs.* SHE	[17]
Mn^2+^/Mn^3+^	0.3	1.51 *vs.* SHE	[18]
I^3−^/I^−^	5.0	0.54 *vs.* SHE	[42]
VBr_3_/VBr_2_	3.0	−0.26 *vs.* SHE	[29]

## 3. Metal Complexes

The transition metal coordination complex comprises a transition metal center and peripheral ligands. The transition metal ion usually acts as the charge transfer center, while the potential can be tuned with different ligands. In complexes with “redox-non-innocent ligands”, charges can also be stored in ligands in addition to the metal center [45]. Compared to transition metal ions, metal complexes own a considerably larger size than single metals ions, and the crossover problem can be mitigated. With the proper choice of ligand, the redox potential of the complex can be tuned towards the positive or negative direction to meet the requirements of catholyte and anolyte. Moreover, ligands can also help to improve the characteristics, such as solubility, kinetic rates and stability.

The research of metal complexes for RFB can be dated back to the 1980s [10,46,47,48]. Metal complexes of iron, cobalt, vanadium, cerium, chromium and ruthenium with various ligands, such as ethylenediaminetetraacetate (EDTA) [25,47], phenanthroline [10,47], triethanolamine [49] and diethylenetriaminepentaacetic acid (DTPA) [48,50], have been applied in both aqueous and non-aqueous systems. In 1988, Matsuda reported a rechargeable redox battery using tris(2,2′-bipyridine)ruthenium(II) ([Ru(bpy)_3_]^2+^) tetrafluoroborate in a non-aqueous electrolyte [46]. The redox couples [Ru(bpy)_3_]^2+^/[Ru(bpy)_3_]^3+^ and [Ru(bpy)_3_]^+^/[Ru(bpy)_3_]^2+^ lead to a cell voltage of about 2.60 V. A concentration of 0.20 M was demonstrated in a functional flow cell with Et_4_NClO_4_/CH_3_CH as the supporting electrolyte. Later, Chakrabarti *et al*. reported a ruthenium acetylacetonate (Ru(acac)_3_) flow battery in tetraethyl ammonium tetrafluoroborate (TEABF_4_)/CH_3_CN electrolyte. More recently, tris(2,2′-bipyridine)iron, tris(2,2′-bipyridine)nickel [51], vanadium acetylacetonate (V(acac)_3_) [52], chromium acetylacetonate (Cr(acac)_3_) [53,54,55] and manganese acetylacetonate (Mn(acac)_3_) [55] were studied. Among these complexes, V(acac)_3_ attracted the most attention due to its good reversibility and a voltage of 2.20 V–2.60 V in various solvents [56]. V(acac)_3_ shows two different voltammetry peaks, corresponding to the reactions:

(9) $$\text{Cathodic: }V{\left(acac\right)}_{3}-e^{-}↔\;{\left[V{\left(acac\right)}_{3}\right]}^{+}\;0.45\text{ V }vs.\text{ Ag}/{\text{Ag}}^{+}$$

(10) $$\text{Anodic: }V{\left(acac\right)}_{3}+e^{-}↔\;{\left[V{\left(acac\right)}_{3}\right]}^{-}\;-1.76\text{ V }vs.\text{ Ag}/{\text{Ag}}^{+}$$

The reversibility has been studied on various electrode materials, on which fast electron transfer is found in the V(acac)_3_/[V(acac)_3_]^+^ reaction, while a slower rate in the reduction [57]. Shinkle *et al*. studied its solubility in various solvents and supporting electrolyte, finding that the solubility were 0.59 M, 0.54 M, 0.53 M and 0.50 M in CH_3_CN, dimethylformamide (DMF), tetrahydrofuran (THF) and dimethyl carbonate (DMC), respectively [58]. On the other hand, Herr *et al*. reported that the solubility of V(acac)_3_ could be 0.80 M, 0.40 M, 0.25 M and 0.30 M in 1,3-dioxolane, THF, dimethyl sulfoxide (DMSO) and acetylacetone, respectively [56]. However, in the reported works, only a maximally 0.05 M concentration of V(acac)_3_ was demonstrated in the charge/discharge tests, making the energy density far too low for real applications. Meanwhile, V(acac)_3_ was reported to be sensitive to moisture and oxygen [59]. Further studies are required to enhance the solubility and stability of V(acac)_3_ before it can be applied in large-scale energy storage systems.

Metallocenes, with a transition metal center sandwiched by two cyclopentadienyl groups, are usually stable and robust [60]. Hwang *et al*. reported a flow battery with ferrocene derivatives in the catholyte and cobaltocene derivatives in the anolyte. With bromoferrocene in the catholyte and bis(pentamethylcyclopentadienyl)cobalt in the anolyte, a discharge voltage of 2.20 V could be achieved [61]. In particular, ferrocene has been used as quasi-reference and overcharge protection shuttles due to its good stability and high electrochemical reaction rates [62,63,64]. With a redox potential of 3.20–3.60 V *vs.* Li/Li^+^ in different organic solvents, ferrocene is also a good choice for the catholyte in a redox flow battery. Zhao *et al*. reported a ferrocene/ferrocenium redox couple as the liquid cathode [65]. Fc/Fc^+^ showed a redox potential of 3.60 V *vs.* Li/Li^+^ in DMF solvent, and the solubility was 0.10 M. The solubility of pristine ferrocene is still far too low to be considered as an energy storage species in a redox flow battery.

To solve this problem, Wei *et al*. reported a molecular engineering strategy of modifying the cyclopentadienyl group by adding a tetraalkylammonium pendant arm with a (trifluoromethanesulfonyl)imide (TFSI^−^) counter anion [66]. Such a modification dramatically increased the solubility of ferrocene by 20 times in carbonate electrolyte. The modified ferrocene, which was ferrocenylmethyl dimethyl ethyl ammonium bis(trifluoromethanesulfonyl)imide (Fc1N112-TFSI), had a solubility of 1.70 M in ethylene carbonate (EC)/propylene carbonate (PC)/ethyl methyl carbonate (EMC) and 0.85 M when 1.20 M LiTFSI was added. The tetraalkylammonium group provides a positively-charged moiety, which will be the preferential sites for the carbonate molecules to be around due to the presence of polarizable oxygen atoms in carbonates that are slightly negatively charged. As a result, the interaction between solvent molecules and modified ferrocene is more favorable than pristine ferrocene. The substituent group also shifted the redox potential by +0.23 V. Thanks to the increased solubility and redox potential, a volumetric energy density of about 50 Wh·L^−1^ was achieved. However, the high concentration catholyte delivered lower voltage efficiency, probably because of the higher viscosity of the solution. The higher concentration also had lower Coulombic efficiency due to the more severe self-discharge.

Cappillino *et al*. reported a metal complex, tris(mnt)vanadium, (mnt = (NC)_2_C_2_S_2_^2−^)), with redox non-innocent ligands. Charges were stored in ligands in addition to the metal center, and as a result, redox non-innocent ligands could also contribute in the charge storage and transfer. This work presented a strong dependence of the redox potential of tris(mnt)vanadium on the cations used in the supporting electrolyte. This unique feature may provide a convenient method to tune the redox potential by varying the supporting electrolyte.

## 4. Metal-Free Organic Compounds

Metal-centered complexes are potentially limited by the scarcity of metal elements. The toxicity of some metals, such as vanadium and chromium, also raises concerns. Metal-free compounds do not require any redox-active metals, representing a promising direction for reducing cost. [67]. Metal-free organic compounds also capitalize on the advantages of natural abundance, structural diversity, potential and solubility tunability and eco-friendliness.

Quinones are especially attractive for redox flow battery applications due to their reversible and fast proton-coupled electron transfer processes. Quinones have been used as solid active electrode materials and overcharge protection mediators in batteries [68,69,70]. Although good electrochemical properties of quinones have been known for many years, limited studies were carried out to employ such molecules in redox flow batteries until recent years. Among different quinones with various mother rings, benzoquinones have the highest solubility and potential [71]. In 2009, Xu and Wen reported a battery with 4,5-dihydroxy-1,3 benzenedisulfonate (DHBDS) and 2,5-dihydroxybenzenedisulfonate acid (sulfonic quinol) as the catholyte [72]. These molecules have good reversibility and relatively high potential, which are critical properties for battery applications. Although only a 0.05 M concentration was demonstrated in a lab flow battery, it showed high Coulombic efficiency of 99% in over 100 cycles, indicating that quinones are promising catholyte materials.

In early 2014, Huskinson *et al*. reported a metal-free flow battery based on 9,10-anthraquinone-2,7-disulphonic acid (AQDS) [73]. AQDS gave high solubility in aqueous solvents (over 1.0 M), and its two-electron two-proton reduction/oxidation process could theoretically double the capacity. This molecule presents a redox potential of 0.23 V *vs.* SHE (with 1.0 M H_2_SO_4_). When coupled with bromine catholyte, the flow battery could deliver a cell voltage of 0.86 V and an energy density of about 50 Wh·L^−1^.

(11) $$\text{Cathodic: }Br_{2}+2e^{-}↔2Br^{-}\;1.09\text{ V }vs.\text{ SHE}$$

(12) $$\text{Anodic: }2AQDS^{-}+2H^{+}+2e^{-}↔2AQDSH\;0.23\text{ V }vs.\text{ SHE}$$

Yang *et al*. reported an organic redox flow battery with quinone derivatives in both the cathodic and anodic sides [74]. 1,2-benzoquinone-3,5-disulfonic acid (BQDS) was used in the catholyte, while AQDS or anthraquinone-2-sulfonic acid (AQS) was used in the anolyte. BQDS has a redox potential at 0.45 V *vs.* mercury-mercurous sulfate electrode (MSE), while the AQS and AQDS show −0.52 and −0.60 V *vs.* MSE, respectively, delivering a cell voltage of about 1.00 V. The researchers reported 1.70 M solubility of these molecules in water, although only 0.20 M was demonstrated in flow cells. More recently, Lin *et al*. reported an aqueous redox flow battery with 2,6-dihydroxyanthraquinone (2,6-DHAQ) as the anodic redox species. For the cathodic part, the bromine was replaced by K_4_Fe(CN)_6_ [75]. The cell was demonstrated with 0.5 M 2,6-DHAQ and 0.4 M K_4_Fe(CN)_6_ and showed an OCV of 1.2 V at 50% state of charge SOC. The use of quinones and ferrocyanide in catholyte provides great advantages over bromine, because they are noncorrosive and less volatile.

The attempt of applying quinone-based molecules in non-aqueous systems is hindered by the poor solubility (less than 0.05 M) of quinone in most non-aqueous electrolytes. In order to solve this problem, Wang and Xu modified the structure of anthraquinone to improve its solubility in non-aqueous electrolyte [76]. Two triethylene glycol monomethyl ether groups were added into the anthraquinone molecular structure, and the solubility was largely improved (0.25 M in LiPF_6_/PC). Unlike the aforementioned quinone-based molecules, in which the two-electron process takes place at the same potential in aqueous electrolyte, this modified anthraquinone presented two well-separated cyclic voltammetry (CV) peaks (2.03 V and 2.27 V *vs.* Li/Li^+^ for the reduction, and 2.47 V and 2.79 V *vs.* Li/Li^+^ for the oxidation) in carbonate solvent. Such a big difference between the oxidative and reductive peaks (about 0.5 V) also indicated the high polarization of this molecule during charge and discharge processes.

Other than quinones, Brushett *et al*. reported an all-organic non-aqueous flow battery based on aromatic molecules [77]. 2,5-Di-*tert*-butyl-1,4-bis(2-methoxyethoxy) benzene (DBBB) was employed in the catholyte. For the anolyte side, quinoxaline-derivative 2,3,6-trimethylquinoxaline (TMeQ) was used [78]. The cell delivered a discharge voltage at 1.30–1.70 V. It is worth pointing out that only a 0.05 M concentration (for both molecules) was used in the cell test, despite much higher solubility being reported for both molecules. Later Huang *et al*. reported a liquid form molecule based on the modification of DBBB by incorporation of polyethylene oxide (PEO) chains with the dimethoxy-di-*tert*-butyl-benzene-based redox structure [79]. The new molecules present reversible redox reactions at around 4.00 V *vs.* Li/Li^+^. In a more recent work, a new redox molecule 3,7-bis(trifluoromethyl)-*N*-ethylphenothiazine (BCF3EPT) was studied as the catholyte species [80]. The radical cation of BCF3EPT showed better stability than the radical cation of DBBB. More importantly, BCF3EPT had higher solubility in PC (1.20 M) than that of DBBB (0.20 M), and a battery consisting of 0.35 M catholyte was successfully demonstrated. On the other hand, Wei *et al*. reported 9-fluorenone and 2,5-di-*tert*-butyl-1-methoxy-4-[2′-methoxyethoxy]benzene (DBMMB) as the anolyte and catholyte redox species [81]. The solubility limit for both 9-fluorenone and DBMMB was 0.90 M in TEA-TFSI/CH_3_CN, while 0.50 M was demonstrated in the battery test. With a redox potential of −1.64 V *vs.* Ag/Ag^+^ for FL and 0.73 V *vs.* Ag/Ag^+^ for DBMMB, this system delivered a voltage of 2.37 V. For these redox species, both the anolyte and catholyte reactions involve free radicals, so the selection of electrolyte is critical for the stability of radical ions.

2,2,6,6-tetramethyl-1-piperidinyloxy (TEMPO) owns quasi-reversible oxidation/reduction electrochemical properties. The TEMPO radical is protected by the resonance structures over the radical center and the alicyclic-nitroxyl structures around the center, which makes it robust in batteries for a sufficiently long time [82]. Previously, TEMPO and TEMPO-based polyradicals have been studied as cathode active materials in rechargeable batteries [83]. TEMPO has also been studied as a charge catalyst for a Li-O_2_ battery and a Mg-O_2_ battery [82,84,85,86,87]. In 2014, Wei *et al*. reported a high concentration TEMPO solution in EC/PC/EMC solvent [88]. The solubility of TEMPO can be as high as 5.2 M. Even with LiPF_6_ as the supporting electrolyte, the solubility of TEMPO can still achieve 2.0 M. This high solubility might be attributed to the similar polarity between TEMPO and the carbonate solvents. In Wei’s study, the TEMPO solution was coupled with a lithium-graphite anode, forming a hybrid flow battery. Due to the high concentration (2.0 M) and high potential (3.50 V *vs.* Li/Li), the flow cell delivered an energy density of 126 Wh·L^−1^, about five-times that of the aqueous all-vanadium flow battery. However, the high concentration causes relatively high viscosity. On the other hand, ionic liquid is an attractive approach to achieve high energy density, because it maximized the solubility of active species. Takechi *et al*. reported TEMPO-based ionic liquid as the catholyte for a redox flow battery [89]. Instead of non-substituted TEMPO molecules, this study selected 4-methoxy-2,2,6,6-tetra-methylpiperidine 1-oxyl (MT or MeO-TEMPO) as a redox compound. When MeO-TEMPO was mixed with lithium bis(trifluoromethanesulfonyl) imide (LITFSI), the mixture exhibited a self-melting behavior and formed a “smooth viscous liquid”. This liquid was stable over a wide temperature range. The MeO-TEMPO/LITFSI (1/1) + H_2_O (17 wt %) was recognized as having well-balanced energy density and viscosity, which had over a 2.0 M concentration and a 72 mPa·s viscosity. A summary of metal complexes and metal-free organic compounds is shown in Table 2.

**Table 2:** Summary of metal complexes and metal-free organic compounds. acac, acetylacetonate; Fc1N112-TFSI, ferrocenylmethyl dimethyl ethyl ammonium bis(trifluoromethanesulfonyl)imide; DME, 1,2-dimethoxyethane.

Redox Species	Demonstrated Concentration/M	Potential/V	Electrolyte	Reference
**[Fe(bpy)_3_]^2+^/[Fe(bpy)_3_]^3+^**	0.40	1.45 *vs.* Ag/Ag^+^	0.5 mol·L^−1^ TEABF_4_/PC	[46]
**[Fe(bpy)_3_]^+^/[Fe(bpy)_3_]^2+^**	0.20	−1.12 *vs.* Ag/Ag^+^		
**V(acac)_3_/[V(acac)_3_]^+^**	0.05	0.45 *vs.* Ag/Ag^+^	TBAPF_4_ in various solvents	[52,56,57,58,59]
**V(acac)_3_/[V(acac)_3_]^−^**	0.05	−1.75 *vs.* Ag/Ag^+^		
**Cr(acac)_3_/[Cr(acac)_3_]^+^**	0.05	1.20 *vs.* Ag/Ag^+^	0.5 mol·L^−1^ TEABF_4_/CH_3_CN	[54]
**Cr(acac)_3_/[Cr(acac)_3_]^−^**	0.05	−2.20 *vs.* Ag/Ag^+^		
**Mn(acac)_3_/[Mn(acac)_3_]^+^**	0.05	0.70 *vs.* Ag/Ag^+^	0.5 mol·L^−1^ TEABF_4/_CH_3_CN	[55]
**Mn(acac)_3_/[Mn(acac)_3_]^−^**	0.05	−0.40 *vs.* Ag/Ag^+^		
**Ru(acac)_3_/[Ru(acac)_3_]^+^**	0.002	1.17 *vs.* Ag/Ag^+^	1 mol·L^−1^ TEABF_4_/CH_3_CN	[53]
**Ru(acac)_3_/[Ru(acac)_3_]^+^**	0.002	−0.60 *vs.* Ag/Ag^+^		
**[Fe(bpy)_3_]^2+^/[Fe(bpy)_3_]^3+^**	0.40	0.50 *vs.* Ag/Ag^+^	0.5 mol·L^−1^ Tetrabutylammonium hexafluorophosphate (TEAPF_6_)/CH_3_CN	[53]
**[Fe(bpy)_3_]^+^/[Fe(bpy)_3_]^2+^**	0.40	−1.95 *vs.* Ag/Ag^+^		
**[Ni(bpy)_3_]^2+^/[Ni(bpy)_3_]^3+^**	0.20	−1.70 *vs.* Ag/Ag^+^	0.5 mol·L^−1^ TEABF_4_/PC	[97]
**Fc/Fc^+^**	0.10	3.20–3.60 *vs.* Li/Li^+^	Various electrolytes	[61,65]
**CoCp_2_/CoCp_2_^+^**	0.01	−1.29 *vs.* Ag/Ag^+^	1 mol·L^−1^ TEAPF_6_/CH_3_CN	[61]
**FcBr/FcBr^+^**	0.01	0.26 *vs.* Ag/Ag^+^		
Bis(pentamethylcyclopentadienyl)cobalt (CoCp*_2_/CoCp*_2_^+^)	0.01	−1.83 *vs.* Ag/Ag^+^		
**Auinoxaline-derivative 2,3,6-trimethylquinoxaline (TMeQ/TMeQ^+^)**	0.05	2.30 *vs.* Li/Li^+^	0.2 mol·L^−1^ LiBF_4_/PC	[77]
**3,7-bis(trifluoromethyl)-*N*-ethylphenothiazine (BCF_3_EPT/BCF_3_EPT)**	0.35	3.90 and 4.40 *vs*. Li/Li^+^	0.2 mol·L^−1^ LiBF_4_/PC	[80]
**Fc1N112-TFSI**	0.80	3.49 *vs.* Li/Li^+^	1 mol·L^−1^ LiTFSI in EC/PC/EMC	[66]
**2,2,6,6-Tetramethylpiperidine-1-oxyl (TEMPO)**	2.00	3.50 *vs.* Li/Li^+^	2.3 mol·L^−1^ LiPF_6_ in EC/PC/EMC	[88]
**4-Methoxy-2,2,6,6-tetra-methylpiperidine1-oxyl (Meo-TEMPO)**	MeO-TEMPO/LITFSI (1/1) + H_2_O(17% wt)	3.50 *vs.* Li/Li^+^	Ionic liquid	[89]
**9-fluorenone**	0.50	−1.64 *vs.* Ag/Ag^+^	Various lithium salts in DME or CH_3_CN [81]	[81]
**2,5-di-*tert*-Butyl-1-methoxy-4-[2′-methoxyethoxy]benzene (DBMMB)**	0.50	0.73 *vs.* Ag/Ag^+^		
**2,5-di-*tert*-Butyl-1,4-bis(2-methoxyethoxy)benzene (DBBB/DBBB^+^)**	0.05	4.00 *vs.* Li/Li^+^	0.2 mol·L^−1^ LiBF_4_/PC	[77]
**V(mnt)_3_^2−^/V(mnt)_3_^−^**	0.02	−1.41 to −1.25 *vs.* SHE;	Various PF_6_^−^ salts in CH_3_CN	[45]
**V(mnt)_3_^−^/V(mnt)_3_**	0.02	−0.23 *vs.* SHE		
**2,6-dihydroxyanthraquinone (2,6-DHAQ)**	0.50	−0.70 *vs.* SHE	1.0 M KOH/H_2_O	[75]
**K_4_Fe(CN)_6_**	0.4	0.50 *vs.* SHE		

* stands for the deca-methyl groups in the Bis(pentamethylcyclopentadienyl)cobalt structure.

## 5. Polysulfide/Sulfur

Sulfur has drawn considerable attention for its high theoretical capacity, non-toxicity and low cost [90]. The aqueous polysulfide/bromine battery (PSB) utilizes Na_2_S in the anolyte, coupled with NaBr as the catholyte [91]. Na_2_S is both abundant and cost-effective, and it gives high solubility in aqueous solvents. A 2.0 M Na_2_S electrolyte has been demonstrated in redox flow batteries [92,93]. A cell voltage of about 1.36 V is given by such a system. Long chain polysulfides also show good solubility in non-aqueous electrolytes [94]. Yang *et al*. reported a membrane-free Li/polysulfide hybrid flow battery with 1,3-dioxolane (DOL)/1,2-dimethoxyethane (DME) as the solvent [95]. Polysulfide had an ultra-high theoretical solubility of 7.0 M in such an electrolyte. To avoid the formation of solid Li_2_S_2_ and Li_2_S phases, the catholyte was cycled between sulfur and Li_2_S_4_. The proof-of-concept Li/polysulfide battery demonstrated a 5.0 M Li_2_S_8_ catholyte, giving a theoretical energy density of 95 Wh·kg^−1^ and 106 Wh·L^−1^. It is worth noting that the cycling test was conducted in a coin cell without flowing, although the researchers claimed that the chemistry could be applied in flow conditions. Despite the high solubility of polysulfide, solid precipitation due to the insoluble short-chain polysulfides formed during long-term cell cycling still remains a problem [96]. Pan *et al*. investigated the solution chemistry of polysulfides in organic electrolyte [94]. The solubility of short chain polysulfides, like Li_2_S_2_, can be slightly improved by using DMSO/LiTf as the supporting electrolyte.

## 6. Li Metal

Lithium is known to have the highest theoretical specific capacity of 3860 mAh/g and a molar lithium concentration of 76.95 M. Meanwhile, as an anodic electrode, lithium provides lower potential (−3.04 V *vs.* standard hydrogen electrode (SHE)) than most redox species, and it is advantageous to obtain a high cell voltage [98]. Therefore, synergy with Li metal seems to be an attractive way to improve the energy density of redox flow battery systems. In 2011, a lithium hybrid flow battery with a lithium metal anode and an aqueous flow catholyte was reported by Goodenough’s group [99,100]. In the demonstrated battery, 0.1 M K_3_Fe(CN)_6_ solution was used as the catholyte. Despite the moderate concentration of catholyte, the hybrid flow battery presented a high cell voltage of 3.40 V, which was much higher than conventional aqueous flow batteries. A similar system was also proposed by Zhou and coworkers, and Fe^3+^/Fe^2+^ was employed as the cathodic redox couple. The electrode reactions of hybrid flow batteries can be described as follows:

(13) $$\text{Cathodic: }M^{+}+{\text{e}}^{-}\;↔M$$

(14) $$\text{Anodic: Li}-{\text{e}}^{-}↔{\text{Li}}^{+}$$

**Figure 3:**
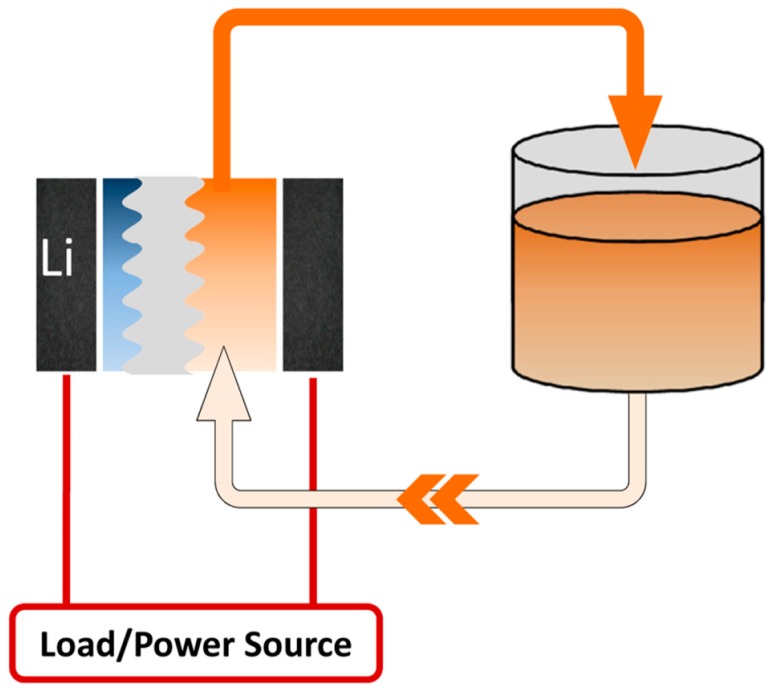
Illustration of a lithium hybrid flow battery. The anodic side consists of lithium metal and organic electrolyte, while the catholyte is circulated.

As shown in Figure 3, the basic setup of a lithium metal hybrid flow battery consists of an anodic compartment with lithium metal in organic electrolyte, a flow through cathode and a solid electrolyte membrane. By combining lithium metal with circulating catholyte, the hybrid flow battery incorporates the advantages of a higher energy density from the lithium battery and independent operation of the power generation and energy storage from the redox flow battery [15].

The key component of the lithium hybrid redox flow battery is the solid electrolyte membrane. In addition to a high Li^+^ conductivity, the membrane is required to block the dendrite growth and redox species crossover. Moreover, the membrane must be mechanically and chemically stable in the cell operation. Currently, the development of a solid electrolyte membrane is still far from being mature. The commercially-available solid electrolyte membranes, including LATP (like Li_1.4_Al_0.4_Ti_1.6_(PO_4_)_3_) and LAGP (like Li_1.5_Al_0.5_Ge_1.5_(PO_4_)_3_), are generally expensive. Under this circumstance, some reports employ a “membrane-free” configuration in non-aqueous systems with a solid electrolyte interface (SEI) layer to protect the lithium metal anode [66,101,102]. Ding *et al*. demonstrated a membrane-free lithium hybrid flow battery with ferrocene/ferrocenium in the catholyte [102]. Due to the fast rate constant of Fc/Fc^+^ and low internal resistance, peak power output reached 4700 W·L^−1^. While the long-term cycling performance is unlikely to be solved, the membrane-free configuration eliminates the need for a solid electrolyte membrane, considerably reducing the cost and allowing for higher power density.

Since the anodic potential and capacity have been fixed by lithium metal, the selection of the catholyte is critical for the overall performance of a lithium hybrid flow battery. High solubility and high redox potential are desirable for catholyte redox species. The reported catholytes have covered various redox couples, ranging from an aqueous system to a non-aqueous system, including transition metal ions, halogen ions [103,104,105,106], metal complexes [65], sulfur/polysulfide, metal-free species, *etc.* Compared to traditional RFBs, the hybrid flow battery with lithium metal anode possesses advantages of a higher operating voltage, a smaller volume and a simple design. Especially, the energy density of the redox flow battery increases drastically by the introduction of a lithium metal anode. However, it should be pointed out that the hybrid configuration loses the flexibility in the anodic compartment, since the lithium metal anode is in static mode rather than flow mode. In addition, the lithium metal anode has problems of poor cycling performance, deleterious dendrite growth and safety concerns.

## 7. Semi-Solid Flow Battery

Duduta *et al*. proposed a semi-solid approach, in which Li-intercalation active materials are circulated in slurries with conductive additives [107]. The main reactions in semi-solid slurries are Li^+^ intercalation and extraction, which is fundamentally different from the abovementioned electrolytes. By employing the active material suspensions rather than the solution, the energy density is no longer limited by the solubility of active species. Solid storage compounds generally contain lithium with a high concentration. For example, the theoretical molar concentration of lithium is about 51.6 M in LiCoO_2_. Although usually only half of the Li^+^ in LiCoO_2_ is usable, the concentration is still considerably high. With the high concentration of solid materials and the wide potential range of non-aqueous solvent, the semi-solid flow battery could be able to deliver high energy density, while keeping the flexibility of the flow battery and avoiding lithium dendrite growth. In Duduta’s report, a semi-solid flow battery that employed LiCoO_2_ (20 vol %, 10.2 M and 1.5% Ketjenblack) as the cathode and Li_4_Ti_5_O_12_ (10 vol %, 2.3 M and 2% Ketjenblack) as the anode was demonstrated.

Later, Hamelet *et al*. applied the semi-solid concept to a silicon suspension flow battery [108]. Silicon is attractive as an anodic active material for its high lithium retention ability, but the large volume change has hindered its development. The semi-solid configuration seems to be suitable to employ silicon, since the volume change will be less problematic in the suspension. Fan *et al*. reported a polysulfide flow battery with a percolating nanoscale conductor network [109]. The polysulfide species are converted reversibly between Li_2_S_8_ and Li_2_S. More recently, Chen *et al*. employed a sulfur-impregnated flow cathode with 20 vol % sulfur and 26 vol % carbon [110]. The volumetric capacity resulting from such a concentration was 12.9 M, leading to a high volumetric capacity, despite the demonstration cell being tested under a very small scale by a syringe pump with 100 μL of electrolyte. Edgar Ventosa *et al*. proposed a non-aqueous semi-solid flow battery based on Na^+^ chemistry using Na_x_Ni_0.22_Co_0.11_Mn_0.66_O_2_ and NaTi_2_(PO_4_)_3_ as the positive and negative electrodes, respectively [111]. The semi-solid flow battery has also been applied in an aqueous system with LiTi(PO_4_)_3_ and LiFePO_4_ as the active materials [112]. Very recently, Janoschka *et al*. reported an aqueous flow battery with micro-molecular polymers as the redox species [113]. By using a dialysis membrane, the redox-active polymers, which have a hydrodynamic radius of around 2 nm, could be effectively charged/discharged in the flow battery.

The semi-solid flow battery provides a promising approach towards higher energy density, while keeping the configuration of decoupled energy and power. However, since the active materials are mostly not electronic conductive, carbon materials need to be dispersed into the slurry to facilitate electron transfer and, thus, inevitably compromise the energy density. Moreover, the electronic conductivity of the suspension network is about 100-times lower than the ionic conductivity and is expected to be the rate-limiting step [107]. Another major concern is the high viscosity that ensues from the increasing solid load. Although this problem can be alleviated by tailoring the interaction between suspension particles, the active materials’ fraction is still limited within around 20 vol % in most reports [114]. The increase in the solid fraction also results in the decrease of ionic conductivity [107]. Given these limitations, the high energy density of the solid active materials may not be fully realized in the semi-solid configuration.

## 8. Redox Flow Lithium Ion Battery

**Figure 4:**
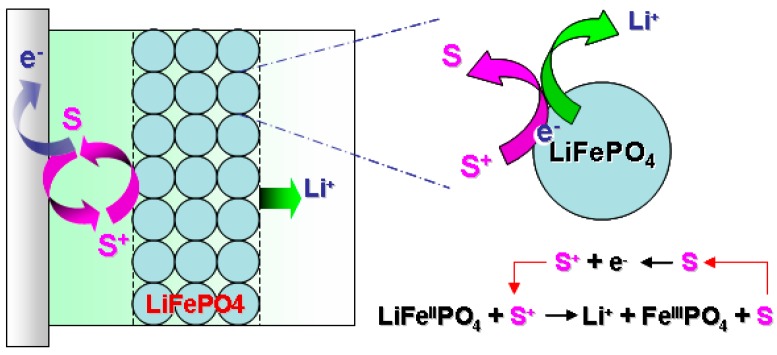
The principle of redox targeting of an insulating electrode material, such as LiFePO_4_, by a freely-diffusing molecular shuttle, S [115] (reprinted with permission from *Angew Chem Int Ed Engl* 45, 8197–8200, (2006); Copyright 2006, WILEY-VCH Verlag GmbH & Co. KGaA, Weinheim).

**Figure 5:**
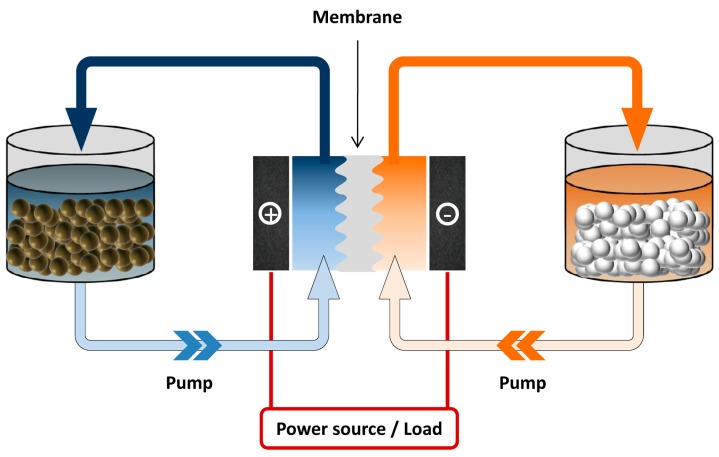
Illustration of a redox flow lithium ion battery. Solid active materials are stored statically in the tanks, while redox molecules are circulated with electrolyte fluids.

The redox targeting concept, which was first proposed by Wang *et al*. in 2006, allows the solid active materials to be charged/discharged without any conductive additives [115] (Figure 4). In 2013, Huang *et al*. reported a redox flow lithium battery (RFLB) based on redox targeting [116]. Unlike the semi-solid configuration, RFLB holds the solid material statically in the tank, and the redox shuttle molecules are circulated in the flow system (Figure 5). The electron transfer between the solid materials and current collector was conducted via the circulation of shuttle molecules. In Huang’s work, ferrocene (Fc) and dibromoferrocene (FcBr_2_) were selected as the reduction and oxidation agents. LiFePO_4_ typically shows a potential of 3.45 V *vs.* Li/Li^+^, while Fc is at 3.25 V and FcBr_2_ is at 3.65 V. As a result, the charge and discharge processes could be achieved by the redox targeting reactions between LiFePO_4_ and shuttle molecules.

Charging process:

(15) $${\text{FcBr}}_{2}\to {{\text{FcBr}}_{2}}^{+}+{\text{e}}^{-}\;\left(\text{on electrode}\right)$$

(16) $${{\text{FcBr}}_{2}}^{+}+{\text{LiFePO}}_{4}\to {\text{FcBr}}_{2}+{\text{FePO}}_{4}+{\text{Li}}^{+}\;\left(\text{in tank}\right)$$

Discharging process:

(17) $${\text{Fc}}^{+}+{\text{e}}^{-}\to \text{Fc }\left(\text{on electrode}\right)$$

(18) $$\text{Fc}+{\text{ FePO}}_{4}+{\text{Li}}^{+}\to {\text{Fc }}^{+}+{\text{LiFePO}}_{4}$$

On the other hand, Pan *et al*. studied the redox targeting for the anodic side of RFLB [117]. Anatase TiO_2_ (1.80 V *vs.* Li/Li^+^), cobaltocene (1.95 V *vs.* Li/Li^+^) and bis(pentamethylcyclopentadienyl)cobalt (1.36 V *vs.* Li/Li^+^) were selected as the solid materials and shuttle molecules.

Discharging process:

(19) $$x{\text{Li}}^{+}+{\text{TiO}}_{2}+x{{\text{CoCp}}^{*}}_{2}\to {\text{Li}}_{x}{\text{TiO}}_{2}+x{{{\text{CoCp}}^{*}}_{2}}^{+}\;\left(\text{in tank}\right)$$

(20) $${{{\text{CoCp}}^{*}}_{2}}^{+}+{\text{e}}^{-}\to {{\text{CoCp}}^{*}}_{2}\;\left(\text{on electrode}\right)$$

Charging process:

(21) $${\text{CoCp}}_{2}\to {{\text{CoCp}}_{2}}^{+}+{\text{e}}^{-}\;\left(\text{on electrode}\right)$$

(22) $${\text{Li}}_{x}{\text{TiO}}_{2}+x{{\text{CoCp}}_{2}}^{+}\to x{\text{Li}}^{+}+{\text{ TiO}}_{2}+x{\text{CoCp}}_{2}\;\left(\text{in tank}\right)$$

Because the solid materials are immobile, the viscosity will not be a concern in RFLB. Additionally, no conducting additive is required in the charge/discharge process. As a result, a large fraction of the solid active materials is allowed in the storage tank. The molar concentration of lithium in LiFePO_4_ is 22.0 M, while the concentration in Li_0.5_TiO_2_ is 22.5 M. If considering a porosity of 50% in the solid materials, the effective concentration could be above 11.0 M. Meanwhile, since most of the energy is stored in the solid materials in the tank, only a diluted concentration of shuttle molecules is needed to conduct the electrons, such as 20.0 mM reported in Huang’s work and 5.0 mM in Pan’s work. A low molecule concentration is meaningful to reduce the crossover contamination and to extend battery life. When TiO_2_ and LiFePO_4_ are combined in the full cell of RFLB, we can expect a cell voltage of about 1.65 V.

## 9. Conclusions and Perspective

Enhancing energy density is one of the most pressing issues in the development of redox flow batteries. As reviewed, recent research on RFB has proposed a variety of approaches towards higher energy density. Particularly, novel redox species have been explored to improve the concentration and cell voltage for both aqueous and non-aqueous systems. Meanwhile, integrating lithium storage materials with redox flow battery is a promising approach to unprecedentedly improve the energy density of RFB. However, some critical challenges still remain before a viable redox flow system is developed.

Although numerous redox species are proposed in non-aqueous electrolytes, most of them are focused on the catholyte, and the anodic species are fairly limited. As a result, the anolyte generally limits the energy density of the whole system. Meanwhile, the redox potential of the anolyte should be pushed lower to maximize the cell voltage. To fabricate a non-aqueous RFB with high energy density, the development of anodic species is urgently needed.

While a hybrid flow battery with a Li metal anode substantially enhances the overall energy density, the flexibility is unfortunately sacrificed in the anode. In addition, the poor cycling stability of the lithium metal anode could be a critical problem of the lithium hybrid flow battery. The cycling stability of lithium metal is deteriorated by dendritic morphology formation during lithium deposition, as well as side reactions between lithium and the electrolyte, which result in low Coulombic efficiency during repeated charging and discharging. The dendrite growth on the metal anode also raises great safety concerns. To address these problems, an anolyte with low potential and high capacity is required to replace the metal anode.

Filling the storage tanks with solid/semi-solid active materials is an effective way to increase the energy density. The redox-targeting concept provides an elegant way for the electron transfer between active materials and current collectors. With the assistance of redox shuttle molecules, the solid active materials could be reversibly charged/discharged while remaining static in tanks. This concept is very promising towards higher energy density. To increase cell voltage, higher potential solid materials, like LiCoO_2_, LiMnPO_4_ and LiVPO_4_F, should be employed in the cathodic side; and accordingly, lower potential solid materials, like Li_4_Ti_5_O_12_, will be suitable for the anodic side. To reduce the overpotential loss, redox molecules matching the potentials of solid materials should also be explored.
